# An interregional measles outbreak in Spain with nosocomial transmission, November 2017 to July 2018

**DOI:** 10.2807/1560-7917.ES.2023.28.17.2200634

**Published:** 2023-04-27

**Authors:** Despina Pampaka, Noemí López-Perea, Aurora Fernández-García, Isabel Huertas-Zarco, Maite Castellanos-Martínez, Katja Villatoro-Bongiorno, Javier Roig-Sena, Nuria Torner, María Mar Mosquera, Juan Emilio Echevarría, Joaquim Ferras Prats, Josefa Masa-Calles

**Affiliations:** 1 National Centre for Epidemiology, Instituto de Salud Carlos III, Madrid, Spain; 2 European Programme for Intervention Epidemiology Training (EPIET), European Centre for Disease Prevention and Control (ECDC), Stockholm, Sweden; 3 Spanish Consortium for Research in Epidemiology and Public Health (CIBERESP), Instituto de Salud Carlos III, Madrid, Spain; 4 National Centre for Microbiology, Instituto de Salud Carlos III, Madrid, Spain; 5 Servei de Vigilància i Control Epidemiològic, Conselleria de Sanitat Universal i Salut Pública, Comunitat Valenciana, Valencia, Spain; 6 Universitat de Barcelona, Barcelona, Spain; 7 Microbiology Department, Hospital Clínic de Barcelona, Barcelona, Spain; 8 Public Health Agency of Catalonia, Generalitat of Catalonia, Barcelona, Spain

**Keywords:** measles, Outbreak, nosocomial transmission, Healthcare workers, Waning immunity, Spain

## Abstract

Given sustained high vaccination coverage and enhanced surveillance for measles, Spain has been free of endemic measles transmission since 2014, achieving elimination certification from the World Health Organization in 2017. In November 2017, measles was introduced through an imported case travelling to the Valencian Community, causing an interregional outbreak. Here, we describe the outbreak using data reported to the national epidemiological surveillance network. The outbreak involved 154 cases (67 males, 87 females) notified in four regions; 148 were laboratory-confirmed and six epidemiologically linked. Most cases were adults aged 30–39 (n = 62, 40.3%) years. Sixty-two cases were hospitalised (40.3%) and 35 presented complications (22.7%). Two thirds of the cases (n = 102) were unvaccinated including 11 infants (≤ 1 year) not yet eligible for vaccination. The main route of transmission was nosocomial; at least six healthcare facilities and 41 healthcare workers and support personnel were affected. Sequencing of the viral nucleoprotein C-terminus (N450) identified genotype B3, belonging to the circulating MVs/Dublin.IRL/8.16-variant. Control measures were implemented, and the outbreak was contained in July 2018. The outbreak highlighted that raising awareness about measles and improving the vaccination coverage in under-vaccinated subgroups and personnel of healthcare facilities are key measures for prevention of future outbreaks.

**Key public health message**

**What did you want to address in this study?**

Measles is a highly contagious viral disease typically characterised by a maculo-papular rash, fever, cough, coryza and conjunctivitis and can lead to serious complications, even death. In Europe, the immunisation programmes recommend two doses of measles-mumps-rubella (MMR) vaccine in early childhood. We wanted to describe an interregional outbreak of measles in Spain after the country gained measles elimination status in 2017.

**What have we learnt from this study?**

We found that the main route of transmission was within the hospital setting, and the outbreak spread to several healthcare facilities, affecting patients, visitors and staff. Not all healthcare staff were vaccinated, although some of them had received two doses of measles vaccine. Almost one in five cases presented so-called modified measles, i.e. they did not have all the typical symptoms of measles.

**What are the implications of your findings for public health?**

Despite high MMR coverage, the risk of outbreaks remains because of under-vaccinated subgroups and importation. In the healthcare setting, monitoring the measles immunity of healthcare staff and support personnel and implementing infection control measures in waiting rooms and emergency departments are important. During measles outbreaks in the post-elimination era, patients with atypical clinical presentation should be tested, independent of their vaccination status.

## Background

Measles is a highly contagious epidemic-prone disease caused by measles virus (MeV; family *Paramyxoviridae,* genus *Morbillivirus*). The disease is typically characterised by a maculopapular rash, fever, cough, coryza and conjunctivitis and can lead to serious complications, even death [1]. The use of effective vaccines against measles has resulted in a decrease in the disease incidence, morbidity and mortality globally. In Spain, a single-dose measles-mumps-rubella (MMR) vaccine was added in the national immunisation programme in 1981 for infants at 15 months of age [2]. Since 2012, a first dose is recommended to be administered to children at 12 months and a second at 3–4 years of age. The national vaccination coverage for the first dose of MMR has been maintained above 95% for more than 20 years and, according to the latest published data, the coverage in 2020 was 95.4% for the first dose and 91.2% for the second [3]. 

Following the 1998 recommendations of the World Health Organization Regional Office for Europe (WHO/Europe) for measles elimination in each country of the European Region [4], Spain prepared a national plan for the elimination of measles [2,5]. The plan was approved in 2000. As a result of sustained high vaccination coverage and enhanced disease surveillance, Spain was able to provide documented evidence for interruption of endemic MeV transmission for 36 consecutive months [6], and thereby gained its measles elimination certification in 2017. Maintaining the elimination status is challenging, as the risk of importation and outbreaks among susceptible pockets of the population remains, especially in a context of resurgence of measles across Europe [7]. In addition, in countries that have eliminated measles, nosocomial spread is an important mode of transmission and a notable threat because of a lower suspicion for measles among healthcare workers and delays in detecting measles cases [8].

### Outbreak detection

In November 2017, an adolescent with measles presentation sought care at a hospital in Valencia, Spain. They had travelled from Romania, where the MeV MVs/Dublin.IRL/8.16[B3]-variant was widely circulating, to Valencia by public transport with a family member. Upon laboratory confirmation of measles 2 days later, the public health authorities were notified and an epidemiological investigation began. Within 10 days, two more cases were notified: a family member of the index case and an infant who was at the same paediatric unit as the index case. Thereafter, a measles outbreak was declared in the Valencian Community. The outbreak lasted for 8 months and included 154 cases and spread to the nearby region of Catalonia. Cases were also reported in two other regions: Madrid and Asturias.

In this report, we describe this interregional outbreak using data reported to the National Epidemiological Surveillance Network (RENAVE in Spanish). We explore the role of nosocomial infections to the magnitude of this outbreak and present the control measures implemented, as well as the lessons learnt.

## Methods

### Measles surveillance

Measles is a mandatory notifiable disease in Spain. Any suspected case of measles is notified to local public health services, which are responsible for case investigation, samples collection, control measures and reporting to regional public health services. The regional public health services report the cases to the RENAVE and complete a standardised case questionnaire via the Spanish surveillance system electronic platform (SiViES).

### Case definition and classification

The RENAVE uses the European Union case definition for measles [9,10]. The clinical criteria include fever (> 38 °C) and maculopapular rash and at least one of the following symptoms: cough, coryza or conjunctivitis. The laboratory criteria include any of the following: (i) MeV-specific antibody response (IgM or IgG seroconversion) in serum or saliva, (ii) detection of MeV nucleic acid, (iii) isolation of MeV or (iv) detection of MeV antigen. Cases were classified as possible cases if they only met the clinical criteria. Probable cases were those who met the clinical criteria and had an epidemiological link to a laboratory-confirmed case. Confirmed cases were defined as those who had not been vaccinated recently (between 7 days and 8 weeks) and met the clinical and laboratory criteria. A measles outbreak was defined as two or more confirmed cases who are temporally related (with dates of rash onset occurring between 7 and 18 days apart), and epidemiologically or virologically linked or both.

To improve the sensitivity of the case definition, we included modified measles, as suggested elsewhere [11]. Cases with atypical clinical presentation who did not meet all clinical criteria but met the laboratory criteria were classified as confirmed cases.

### Laboratory diagnosis and genomic analysis

Clinical specimens, i.e. serum for serology and throat swab or urine for molecular detection, were submitted to local or regional laboratories for laboratory investigation of measles [12]. In addition, most of the samples from confirmed cases were sent for genotyping to the national reference laboratory for measles and rubella at the National Centre for Microbiology at Instituto de Salud Carlos III in Madrid; the samples from cases reported in Catalonia were sent to the Catalonia regional reference laboratory for measles and rubella. 

For molecular analysis, the 450 nucleotides that encode the C-terminus of the viral nucleoprotein (N450), defined by WHO for genotyping, were amplified and sequenced according to a protocol previously described [13]. Sequences were edited using BioEdit v.7.2.5 and aligned with MAFFT v.7 software. Every N450 sequence was named in accordance with the WHO´s standard nomenclature and deposited in the WHO Measles Virus Nucleotide Surveillance (MeaNS) database [14]. The MeaNS tools for genotyping and searching for identical sequences were used to assign a genotype and N450 sequence variant or ‘named strain’. Each set of identical sequences was identified by the name of the earliest sequence for those not linked to any described ‘named strain’ [15]. Phylogenetic analysis was performed by the method of maximum likelihood (ML) using PhyML (http://www.atgc-montpellier.fr/phyml/) with the best evolutionary model previously selected in the model selection tool SMS. The phylogenetic tree was edited using MEGA v.7 software.

### Epidemiological investigation

The local and regional public health services led the epidemiological investigation and the implementation of control measures. They interviewed suspected cases using a standardised measles questionnaire to identify the potential exposure including visits to healthcare centres, to determine links with confirmed cases and to trace contacts. The questionnaire included items on demographic and clinical characteristics, complications, risk factors (e.g. recent travel and vaccination history) and laboratory results [9]. The questionnaires were then completed and submitted to the RENAVE.

### Data analysis

Cumulative incidences (per 100,000 population) per region and per province were computed using population data (1 Jan 2018) from the Spanish National Institute of Statistics (INE) [16]. We calculated frequencies, proportions, medians and interquartile range, according to the type and distribution of the variables. These analyses were performed in Microsoft Excel and in RStudio (Version 1.4.1106) [17].

## Results

Overall, 180 suspected cases linked to this measles outbreak were notified to the RENAVE. Of these, 148 were laboratory-confirmed, six were probable and 26 were excluded, as they did not meet the laboratory criteria for measles. Both laboratory-confirmed and probable cases were considered in our analyses.

### Epidemiological investigation

**Figure 1:**
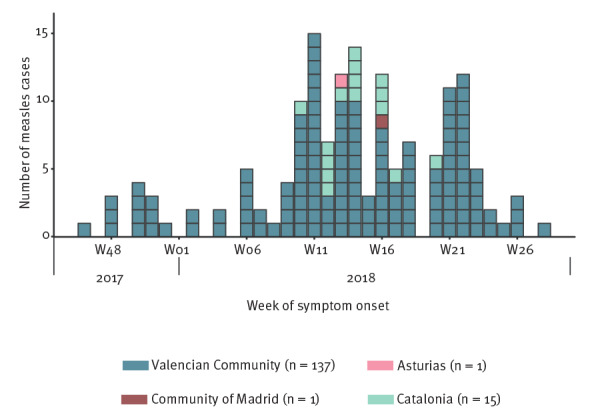
Measles cases by date of symptom onset and autonomous region in an interregional outbreak, Spain, November 2017–July 2018 (n = 154)

The first case had rash onset on mid-November 2017 and the last case on mid-July 2018. Figure 1 shows the evolution of the outbreak in terms of week of symptom onset and region of residence of cases.

The outbreak began in the Valencian Community and spread to neighbouring territories. In the Valencian Community, the provinces affected were Valencia (4.9 cases/100,000 population) and Castellón (2.5 cases/100,000 population). In the region of Catalonia, the province of Tarragona (1.8 cases/100,000 population) had several cases. One case from Asturias (0.1 case/100,000 population) and one case from the Community of Madrid (< 0.1 case/100,000 population) were also reported.

The epidemiological investigation revealed transmission chains with nosocomial, intrafamilial and community transmission. The index case was hospitalised at a healthcare centre (Centre A) 1 day after developing symptoms. Two children, as well as a healthcare worker, were exposed to the case in the hospital’s emergency waiting room at Centre A and developed a rash in the 12 days following. More patients, visitors and healthcare professionals were affected. Other healthcare centres and personnel were consequently affected because of infected patients or infected personnel visiting different health centres within the same region, adding to a total of 108 cases in this chain by mid-July 2018.

Another chain with prominent hospital transmission comprising 14 cases was identified in Tarragona in March 2018. The primary case, who was also identified retrospectively, accompanied a family member to Centre A in Valencia in mid-February 2018, where they were exposed to a measles case. Upon return to Tarragona, the patient developed symptoms later in March 2018 and visited a hospital the following day. This event resulted in the initiation of a transmission chain in that healthcare setting (Centre B); eight cases were hospital staff. 

Another transmission chain with 14 cases was detected in Castellón and involved children and employees at a kindergarten, healthcare-associated cases, as well as community cases. The primary case, who was identified retrospectively, had been hospitalised in Centre B in Valencia. In total, this chain affected six infants and children: of these, three were less than 1 year of age and therefore unvaccinated. The primary case was 1 year old, but vaccination had been postponed because of health problems; the case had unvaccinated siblings under the age of 5 years. 

The outbreak investigation also revealed intrafamilial transmission among unvaccinated members of a Roma community (n = 15 cases) in Valencia. It was not possible to be linked with a confirmed measles case nor a healthcare centre.

The shortest chain including two cases, occurred in a transport setting. A case from Asturias visited Valencia at the time of the outbreak and then travelled with a person from Madrid, who then tested positive for measles. No further cases were identified related to these two cases.

### Transmission settings

Transmission of measles occurred primarily in healthcare centres (n = 65, 42.2%) and affected medical and non-medical personnel, as well as patients and visitors. According to the case investigation forms, cases were mostly exposed in waiting rooms, emergency and paediatric wards. More than a quarter of the cases (n = 41, 26.6%) reported that they were not aware of a contact with a measles case, while 37 (24.0%) were exposed in households. Other settings of transmission included schools, kindergarten, workplaces and means of transport (n = 11, 7.1%).

**Figure 2:**
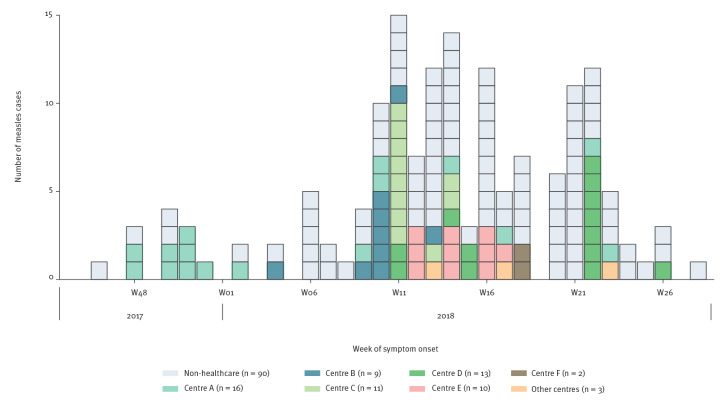
Measles cases by healthcare centre in an interregional measles outbreak, Spain, November 2017–July 2018 (n = 154)

Figure 2 illustrates the spread of the outbreak in the various healthcare centres, according to the exposure setting. More than six centres were affected, and cases related to these centres were reported almost until the end of the outbreak. Spread between centres occurred via healthcare workers, patients and caregivers who visited different centres.

### Characteristics of cases

The main demographic and clinical characteristics of the confirmed and probable cases are summarised in the Table. The median age of cases was 33 years (range: 0–55 years) and 87 were females (56.5%). Eleven cases occurred in infants aged less than 1 year and another 11 in children between 1 and 9 years of age. The greatest proportion of infections (n = 62, 40.3%) was observed among those between 30 and 39 years of age. At least one in four cases (n = 41, 26.6%) worked in a healthcare setting as either a healthcare worker or as support personnel.

**Table:** Characteristics of measles cases of an interregional measles outbreak, Spain, November 2017–July 2018 (n = 154)

Characteristics	Cases	
	n	%
Sex		
Females	87	56.5
Males	67	43.5
Age group (years)		
< 1	11	7.1
1–4	5	3.2
5–9	6	3.9
10–14	2	1.3
15–19	5	3.2
20–29	29	18.8
30–39	62	40.3
40–49	25	16.2
50–59	9	5.8
All, median age (range)	33 (0–55)	
Vaccination status		
0 doses	102	66.3
1 dose	10	6.5
≥ 2 doses	21	13.6
Unknown	21	13.6
Healthcare centre staff		
Yes	41	26.6
No	113	73.4
Measles presentation		
Classic	126	81.8
Modified	28	18.2
Symptoms^a^		
Rash	153	99.4
Fever	149	96.8
Cough	100	64.9
Coryza	64	41.6
Conjunctivitis	51	33.1
Hospitalisation		
Yes	62	40.3
No	92	59.7
Complications		
No complications	101	65.6
Diarrhoea	5	3.2
Pneumonia	14	9.1
Otitis	3	1.9
Other	11	7.1
Unknown	20	13.0

^a^ Multiple symptoms are possible.

There were 28 cases (18.2%) who did not meet the case definition for classic measles and were considered as examples of modified measles (Table). These cases were identified through contact tracing and active case search. All but one case presented rash (n = 153, 99.4%) and the vast majority (n = 149, 96.7%) reported fever. Sixty-two cases (40.3%) were hospitalised and 33 (21.4%) presented complications. The most common complication was pneumonia (n = 14, 9.1%). No deaths were notified.

## Vaccination status

**Figure 3:**
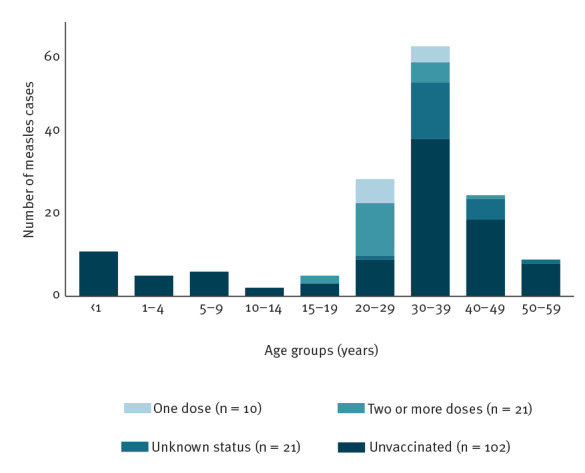
Measles cases by vaccination status and age group in an interregional measles outbreak, Spain, November 2017–July 2018 (n = 154)

Overall, 102 of 133 cases with known vaccination status were unvaccinated (76.7%) and of these, five received a measles-containing vaccine dose for prophylactic purposes after being identified as contacts of confirmed cases. Ten (7.5%) were vaccinated with one dose and 21 (15.8%) had received two doses. The distribution of cases by vaccination status and age group is provided in Figure 3. Among the unvaccinated children aged under 15 years, there were 11 infants aged under 1 year who were not eligible for vaccination. Of the 37 healthcare professionals and support personnel in healthcare facilities with known vaccination status, 20 (54.1%) were unvaccinated, four (10.8%) had received one dose and 13 (35.1%) had received at least two doses.

### Molecular analysis

**Figure 4:**
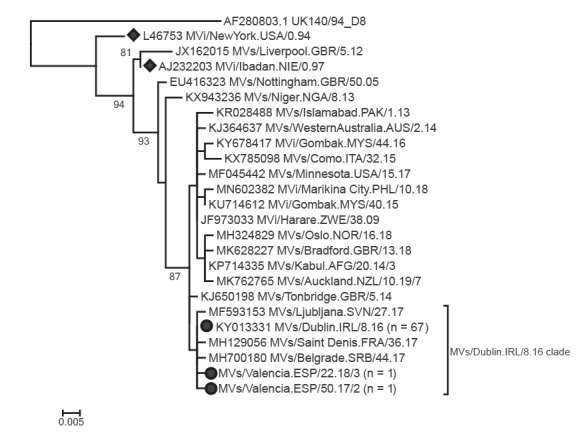
Phylogenetic tree of available measles virus genotype B3 N450 variant sequences with sequences from this outbreak, Spain, November 2017–July 2018

MeV: measles virus; N450: C-terminus of the MeV viral nucleoprotein.

Specimens from all transmission chains were genotyped. Genotype B3 was identified in all specimens analysed (n = 69 cases). Most of the N450 sequences were identical to the MVs/Dublin.IRL/8.16/[B3]-variant (GenBank accession number: KY013331), which was circulating in different European countries at that time and was responsible for an epidemic in Romania [18]. In addition, two N450 sequences showing only one nucleotide mutation compared to the MVs/Dublin.IRL/8.16[B3]-variant were identified: Mvs/Valencia.ESP/50.17/2 (C291T) and MVs/Valencia.ESP/22.18/3 (A83G). All the sequences belonged to the same phylogenetic clade according to the phylogenetic analysis (Figure 4).

Sequences detected in the outbreak (n = 69), Spain (Nov 2017–July 2018) were compared to the variants (named strains) of MeV genotype B3 previously described and deposited in MeaNS (n = 20). Analysis was carried out in PhyML, using TN93 + I as an evolutionary model and bootstrap for the statistical analysis. Bootstrap values above 80 are shown. A MeV D8 genotype reference sequence (AF280803) was used as the outgroup to root the tree. Two MeV genotype B3 references sequences designated by WHO were included (black diamond). The sequences detected in the outbreak are labelled with a black circle. The n equals the number of sequences identified in the outbreak.

## Outbreak control measures

Upon detection of the outbreak, local and regional public health authorities implemented control measures. Cases were advised to stay in isolation for 4 days after the onset of symptoms, and contact tracing was performed to identify susceptible contacts, i.e. those unvaccinated or with unknown vaccination status who had a greater risk of infection. The list of passengers who used the same public transport as the index case was shared with the corresponding autonomous regions.

During contact tracing, the immunisation status of contacts of all cases was assessed and prophylactic MMR vaccine was offered to unvaccinated or partially vaccinated contacts, as well as immunoglobulin where appropriate.

Extensive measures were taken in healthcare settings. The regional public health services published recommendations regarding vaccination of healthcare workers and other personnel and highlighted the importance of complete immunisation. Healthcare services were asked to identify and vaccinate susceptible healthcare workers. Vaccination was provided to susceptible employees, i.e. those born after 1971, without proof of vaccination or laboratory proof of immunity. In some centres, a serological test for measles IgG was offered before vaccination. Healthcare workers who had been in contact with cases and did not receive vaccination were excluded from work for at least 18 days since the day they had contact with the measles case. Information about measles was shared among the healthcare networks to raise awareness about the disease and alert health professionals about the risk of measles transmission, independent of age.

Reports and press releases were prepared and shared with the health community and with the public. The National Center for Epidemiology, the working group who revises the national plan for measles elimination and the epidemiologists at the regional public health authorities shared a weekly report on this outbreak. In August 2018, the outbreak was proclaimed over, after no new cases were identified for two consecutive incubation periods.

## Discussion

We describe an interregional outbreak of measles genotype B3 in Spain, which occurred 5 months after the country gained its elimination status in 2017. The outbreak lasted for 8 months and involved 154 cases, which corresponded to a national cumulative incidence of 0.3 per 100,000 population. Two in five cases required hospitalisation, slightly less than the overall hospitalisation rate in Europe [19]. The outbreak underscored the severity of measles, with fourteen cases of pneumonia reported.

The outbreak that started in Valencia was linked to an imported case from Romania, where an active MVs/Dublin.IRL/8.16[B3]-variant measles epidemic was ongoing at that time [18]; most of the sequences identified in this outbreak were the same strain. Previous work described two transmission chains of this outbreak [20,21]. The outbreak investigation and the extensive contact tracing conducted by the local and regional epidemiological services in the Valencian Community and Catalonia found a clear epidemiological link between the outbreak in Valencia and the transmission chains in Castellón and Tarragona, which was then complemented by the virological and molecular study. The primary cases for the two latter transmission chains visited healthcare facilities in the city of Valencia during the ongoing measles outbreak.

The molecular analysis together with the temporal and spatial data from the epidemiological investigation suggested the existence of a single outbreak. Nevertheless, to differentiate the chains of transmission and possible importations not detected by the epidemiological investigation, the use of more variable genome regions may be valuable. This would provide more resolution in a complementary way for the analysis of N450 variants, given the wide circulation of variants such as MVs/Dublin.IRL/8.16[B3].

The age distribution of cases in an outbreak depends on the immunity of the affected population. Spain has a high childhood vaccination coverage, which is one explanation why cases aged 20 years and above accounted for most cases (81.1%) in this outbreak, while in 2018 in the EU/EEA, the most affected age groups were those below 20 years (64%) [19]. These findings are consistent with the results of the national seroprevalence study that was conducted in 2017–18 [22]. The study showed that participants in the age group 20–29 years (cohorts born between 1988 and 1997) had the lowest seropositive percentage (87%), and the lowest levels of antibodies against measles, suggesting a possible waning effect because of the longer period since vaccination, as other authors have previously described [23], and the absence of contact with the MeV [21,24]. Moreover, during the first years of the implementation of measles vaccination in Spain, the coverage was not high [25], which created immunity gaps among those born close to the implementation; it was only since 1999 that the coverage for the first dose of MMR has been maintained above 95% [26]. The low coverage at the beginning of the implementation, in combination with the lack of vaccination registries, could have also contributed to the relatively high percentage of individuals with unknown vaccination status in the 30–39-year age group. In this outbreak, we also noted an immunity gap among children and among unvaccinated young adults of the Roma community, as has been observed in previous outbreaks in Spain [27,28] and other European countries [29,30]. These findings indicate that there are still susceptible pockets in the population.

Measles occurred among fully vaccinated individuals, as reported elsewhere [23,24,31-34]. In populations with high vaccination coverage, the expected proportion of vaccinated cases is also higher. Moreover, in this study we observed that three in five fully vaccinated cases were healthcare workers with a median age of 27 years. Apart from the waning immunity in those aged 20–39 years, healthcare workers also experience higher levels of exposure to MeV and thus have a higher risk of infection. Of note, none of the vaccinated cases were hospitalised and only one case reported complications, as vaccinated cases usually experience milder disease [23,24,31-34].

Previous reports suggest that infection of vaccinated individuals does not frequently result in secondary cases [23,24,34,35], which can be explained by the lower viral load of breakthrough infections compared with naive infections [21,34]. Albeit rare, secondary transmission to unvaccinated individuals has been observed in situations of intense exposure, such as within the same household [32]. In this outbreak, secondary transmission was noted in at least one chain; an unvaccinated case was infected after travelling with a case vaccinated with a single dose of MMR.

Transmission of MeV in healthcare settings is an emerging public health threat and has become an important mode of transmission, especially in countries where measles has been eliminated [8,26,36]. Frequent nosocomial outbreaks have been reported across Europe [23,37-39]. Cases involved healthcare workers, visitors and patients. Transmission mainly occurred in waiting rooms, emergency and paediatric wards, in line with other outbreaks [40-42].

Mitigating nosocomial transmission as early as possible is crucial, as patients may have an elevated risk for severe disease because of underlying conditions [8]. In addition to the health-related risks for patients, visitors and healthcare workers, the cost of controlling a measles outbreak in a healthcare setting is considerable and absenteeism results in disruption of services [36]. These consequences add to the importance of achieving high two-dose vaccination uptake among healthcare workers. Although vaccination against measles in healthcare workers in Spain is not mandatory, since 2017 the national vaccination guidelines recommend two MMR doses for susceptible professionals [43]. In addition, the updated national plan for the elimination of measles and rubella recommends that individuals working at healthcare facilities, including students, should be vaccinated against measles and rubella [44]. The spread of this protracted outbreak to several facilities highlights the need to implement these guidelines to prevent future outbreaks. Healthcare centres should ensure that all professionals have presumptive evidence of immunity to measles upon employment, routinely monitor their status and organise catch-up vaccinations. These activities should not only target permanent staff but also support personnel who are contracted by other companies [21,39], as well as students and volunteers of non-governmental organisations who work in these settings. 

In addition, it is important that isolation protocols and infection control guidelines be instituted in hospitals, to minimise nosocomial spread of infection. Good practices used in other countries, such as pre-screening over the phone [35], screening in the prodromal phase [45] or isolation in different rooms or even at home, can be adopted. Some of these practices have been successfully implemented in Spain during the COVID-19 pandemic, demonstrating that these preventive measures are feasible. Moreover, hygiene masks can be used in emergency departments and waiting rooms, especially at times of ongoing measles epidemics [8,33]. In this outbreak, we also observed that cases had been infected after visiting multiple healthcare centres seeking medical care while symptomatic, after providing care or accompanying measles cases in their family. Therefore, strategies for raising awareness about measles should include messages for caregivers, parents and patients, especially at times of an ongoing epidemic.

Delays in detecting measles or misdiagnoses because of atypical presentation of the disease could have contributed to the spread of the outbreak. Modified measles symptoms have been reported in vaccinated individuals [11] and have also been observed in Spain over the last years [33]. In this outbreak, approximately one in five cases did not develop cough, coryza or conjunctivitis, but the vast majority (96.1%) presented both rash and fever. Conducting more sensitive investigations during outbreaks, including all patients with febrile rash, has been previously recommended [46]. The use of a more sensitive case definition for modified measles suitable has been applied in outbreaks in communities with high vaccination coverage; this has been discussed elsewhere [11,37].

Our study is subject to some limitations. Firstly, for some cases it was not possible to identify the exposure context. Secondly, some important variables such as vaccination status had missing values. This was observed particularly among those aged 30 years or above because of lack of registers and electronic records, or lack or loss of vaccination cards, which led to self-reported vaccine status in some instances. 

Despite the outbreak, the measles elimination status in Spain was maintained, as the import-related cases did not induce endemic transmission. The high levels of population immunity against measles, the highly effective MMR vaccine, and an effective public health response to reported measles cases limited the size and duration of the outbreak. However, as the risk of measles outbreaks related to importation of the virus remains, vaccination activities should be reinforced for the under-vaccinated subpopulations and the overall coverage must be maintained high to prevent future outbreaks and reduce disease morbidity and mortality.

## Conclusions

This interregional outbreak of measles in Spain occurred after the country gained its elimination status. The outbreak was linked to an imported case and involved 154 cases, mostly young adults (aged 19–40 years) and unvaccinated children (aged under 1 year). Despite the high MMR coverage, there are still under-vaccinated subgroups in the population and the risk of outbreaks due to importation remains. The main route of transmission was nosocomial, and the outbreak spread to several healthcare facilities, affecting patients, visitors and staff. This highlights the importance of implementing the national guidelines for measles immunisation for healthcare professionals and support personnel. In the post-elimination era, delays in diagnosis or misdiagnoses can be prevented by raising awareness about measles and the atypical presentation of the disease.
